# 

**Published:** 1924-08

**Authors:** 


### HOSPITAL and HEALTH REVIEW

*Established 1886* *No.* 1877. *Vol. LXXIII*

*New Series.* Vol. III. No. 35. *August,* 1924. Price Sixpence

CONTENTS

| | PAGE |
|---|---|
| Beginning at the Beginning .. .. .. .. | 227 |
| The Health of the Workless .. .. .. | 228 |
| Notes and Comments .. .. .. .. .. | 229 |
| Hospitals and the Entertainments Duty—"An Agreed Honorarium"—Health in the Factory—A Pillar of Cloud—Wholesome Bread—The Food of the Insane—Work for the Tuberculous—Cancer and Diet—Registration of Nursing Homes—Exercise and Vitamins—"Not Proven"—Clarifying Psycho-Analysis—True and False Vinegar—Old Brisbane Hospital | |
| Hospital Men of Mark: Mr. Thomas Hayes .. | 233 |
| The Citizen of the Future .. .. .. .. .. | 234 |
| A Ward for Indian Seamen .. .. .. .. | 235 |
| Warwickshire's Memorial to King Edward VII. . | 235 |
| The Public Health: Interviews with Local Authorities: Tuberculosis in Lancashire .. | 236 |
| Concerning Memorials. By Theodore Ruete .. | 238 |
| Medical Staffs of Voluntary Hospitals. By C. E. Stewart Flemming, M.R.C.S., L.R.C.P.. .. | 239 |
| A Haven of Peace .. .. .. .. .. .. | 241 |
| National Health Insurance Notes .. .. .. | 242 |
| Correspondence .. .. .. .. .. .. | 243 |
| Exhibition by International Tennis Players .. | 243 |
| Papworth Village Settlement .. .. .. | 244 |
| A Transformed Hospital .. .. .. .. | 246 |
| Hospital Progress and Finance .. .. .. | 247 |
| London School of Hygiene and Tropical Medicine | 249 |
| Prince Tafari at St. Thomas's Hospital .. .. | 249 |
| Lord Dawson on Hospital Problems .. .. | 249 |
| Reviews of Books .. .. .. .. .. | 250 |
| Points from Hospital Reports .. .. .. | 254 |
| Echoes of the Month .. .. .. .. .. | 255 |
| Hospital and Health News .. .. .. .. | 256 |
| Recent Appointments .. .. .. .. .. | 258 |
| Answers to Correspondents .. .. .. .. | 258 |

